# Complete Genome Analysis of a Rabbit Rotavirus Causing Gastroenteritis in a Human Infant

**DOI:** 10.3390/v7020844

**Published:** 2015-02-17

**Authors:** Melisa Berenice Bonica, Mark Zeller, Marc Van Ranst, Jelle Matthijnssens, Elisabeth Heylen

**Affiliations:** KU Leuven-University of Leuven, Department of Microbiology and Immunology, Laboratory for Clinical and Epidemiological Virology, Rega Institute for Medical Research, B-3000 Leuven, Belgium; E-Mails: m.b.bonica@gmail.com (M.B.B.): mark.zeller@uzleuven.be (M.Z.); vanranstmarc@gmail.com (M.V.R.); jelle.matthijnssens@uzleuven.be (J.M.)

**Keywords:** group A rotavirus, rabbit to human interspecies transmission, G3P[14], complete genome characterization

## Abstract

Group A rotaviruses (RVA) are responsible for causing infantile diarrhea both in humans and animals. The molecular characteristics of lapine RVA strains are only studied to a limited extent and so far G3P[14] and G3P[22] were found to be the most common G/P-genotypes. During the 2012-2013 rotavirus season in Belgium, a G3P[14] RVA strain was isolated from stool collected from a two-year-old boy. We investigated whether RVA/Human-wt/BEL/BE5028/2012/G3P[14] is completely of lapine origin or the result of reassortment event(s). Phylogenetic analyses of all gene segments revealed the following genotype constellation: G3-P[14]-I2-R2-C2-M3-A9-N2-T6-E5-H3 and indicated that BE5028 probably represents a rabbit to human interspecies transmission able to cause disease in a human child. Interestingly, BE5028 showed a close evolutionary relationship to RVA/Human-wt/BEL/B4106/2000/G3P[14], another lapine-like strain isolated in a Belgian child in 2000. The phylogenetic analysis of the NSP3 segment suggests the introduction of a bovine(-like) NSP3 into the lapine RVA population in the past 12 years. Sequence analysis of NSP5 revealed a head-to-tail partial duplication, combined with two short insertions and a deletion, indicative of the continuous circulation of this RVA lineage within the rabbit population.

## 1. Introduction

Group A rotaviruses (RVA) are enteric pathogens and a leading cause of severe diarrhea in human infants, as well as in young animals worldwide [1,2]. The rotavirus genome consists of 11 segments of double-stranded RNA encoding six structural viral proteins (VP1 to VP4, VP6, and VP7) and six nonstructural viral proteins (NSP1 to NSP6) [3,4]. The infectious virion consists of three concentric protein layers that surround the  gene segments. VP7 (Glycoprotein) and VP4 (Protease sensitive protein) are the outer capsid proteins involved in a dual-classification system determining the G- and P-genotypes, respectively [2]. To date, RVAs are classified into at least 27 G- and 37 P-genotypes [5,6]. However, a new classification system that takes into account all 11 segments was introduced in 2008. The nomenclature Gx-P[x]-Ix-Rx-Cx-Mx-Ax-Nx-Tx-Ex-Hx represents the genotypes of the VP7-VP4-VP6-VP1-VP2-VP3-NSP1-NSP2-NSP3-NSP4-NSP5 encoding gene segments, respectively [7].

The most common G-genotypes of human RVAs are G1, G2, G3, G4, G9, and G12 while the most common human P-genotypes are P[4], P[6] and P[8]. The most typical G-/P-genotype combinations found in humans are G1P[8], G3P[8], G4P[8], G9[8], G12P[8], and G2P[4], with G1P[8] being the most prevalent worldwide [5,8,9]. For human RVA strains, two major genotype constellations I1-R1-C1-M1-A1-N1-T1-E1-H1 (Wa-like) and I2-R2-C2-M2-A2-N2-T2-E2-H2 (DS-1-like), have been observed. Human Wa-like RVA strains are believed to possess several gene segments that have a common ancestor with porcine RVA strains and are most commonly found in combination with P[8], whereas most gene segments of human DS-1-like strains are believed to have a common ancestor with bovine RVA strains and are mostly found in combination with P[4]. A third (minor) human genotype constellation, I3-R3-C3-M3-A3-N3-T3-E3-H3 (AU-1-like) seems to share a common ancestor with cats or dogs [10]. The P[14] genotype has sporadically been found in humans and is believed to be the result of interspecies transmissions from animals which belong to the mammalian order Artiodactyla, since P[14] strains are described in animals such as goats, antelope, cattle, sheep and guanacos [11,12]. However, it is also a P-genotype frequently found in lapine RVA strains [13,14,15]. Currently, not many studies have focused on rotavirus infections in rabbits in general and their molecular characteristics in particular. Previous studies showed that P[14] and P[22] are the most typical VP4 genotypes found in lapine rotaviruses, with G3 being the most common VP7 genotype [16,17]. The G3 RVA genotype has been described in a wide range of different host species. In fact, it has been isolated in humans, rabbits, pigs, birds, bats, cats, dogs, monkeys, horses, mice, cows, and lambs [13,17,18,19]. Three lapine RVA strains were completely sequenced so far: RVA/Rabbit-tc/ITA/30-96/1996/G3P[14] and RVA/Rabbit-tc/CHN/N5/1992/G3P[14] were found to possess a G3P[14] genotype, whereas RVA/Rabbit-tc/NLD/K1130027/2011/G6P[11] showed a G6P[11] genotype [14,15,20]. However, phylogenetic analyses of all 11 segments of the K1130027 lapine strain indicated that it represented a direct interspecies transmission from a bovine-like RVA strain to a rabbit colony [20] and as a consequence, this strain cannot be considered a typical lapine RVA strain.

Because of the segmented nature of the RVA genome, RVA genetic diversity can be generated through reassortment events, involving one or multiple gene segments [21,22]. Another way of generating genetic diversity is the direct virus transmission from an animal to a human host called interspecies transmission. Interspecies transmission events that result in gastroenteritis are relatively rare [10,23]. In 2000, the first reported case of a lapine G3P[14] RVA strain infecting a human was detected in Belgium [13]. This strain (RVA/Human-wt/BEL/B4106/2000/G3P[14]) was demonstrated to be entirely of lapine origin, but still able to cause severe gastroenteritis in a child [14]. More recently, another zoonotic G3P[14] infection was detected in Australia: strain RVA/Human-wt/AUS/RCH272/2012/G3P[14] was identified in a 12-year-old child hospitalized with gastroenteritis clustering closely to bovine(-like) or thus far unclassified RVA strains [24].

The RVA strain RVA/Human-wt/BEL/BE5028/2012/G3P[14] was isolated from a two-year-old child with gastroenteritis in Belgium. In this study we sequenced and analyzed the complete genome of strain BE5028 with the aim of determining whether this strain represents a direct interspecies transmission or a human-animal reassortant and to what extent BE5028 is related to previously isolated lapine(-like) RVAs.

## 2. Materials and Methods

### 2.1. Rotavirus Strains

The rotavirus sample BE5028 was isolated during routine RVA surveillance in Belgium in July 2012 from a two year old child which was not vaccinated nor hospitalized, but did show symptoms of gastroenteritis, such as diarrhea and vomiting.

### 2.2. RNA Extraction

Viral RNA was extracted from fecal material of the RVA BE5028 strain by using the QIAamp Viral RNA Mini Kit (Qiagen/Westburg, The Netherlands) according to the manufacturer’s instructions.

### 2.3. RT-PCR

The RNA template was denatured for 2 min at 95 °C, and reverse transcriptase PCR (RT-PCR) procedure was carried out by using the Qiagen OneStep RT-PCR Kit (Qiagen/Westburg, The Netherlands). Some primers for the amplification were previously described [13,14], others were developed based on alignments of sequences retrieved from GenBank (Table S1). An initial reverse transcription step was carried out at 50 °C for 30 min, followed by polymerase activation at 95 °C for 15 min, 35 cycles of amplification (denaturation: 30 s at 94 °C; annealing: 30 s at 45 °C (VP4, VP6, VP7, NSP2-NSP5) and at 47 °C (VP1-VP3, NSP1); extension: 6 min at 72 °C for long segments VP1-VP4 and NSP1, and 2 min at 72 °C for short segments VP6, VP7 and NSP2-NSP5), with a final extension at 74 °C for 10 min (long segments) and at 75 °C for 10 min (short segments).

### 2.4. Nucleotide Sequencing

In order to remove unincorporated nucleotides and excess primers from the PCR product, the ExoSAP Clean-up Kit (IsogenLifeScience, Belgium) was used. The PCR amplicons were then sequenced using the BigDye Cycle Sequencing Kit (Applied Biosystems, USA). The sequencing was performed with the same forward primers as was used for the RT-PCR for the short segments, and with both forward and reverse primers for the long segments. Primer walking sequencing was performed to cover the complete gene segment if necessary. After the sequencing reaction, an ethanol precipitation was performed and the final products were loaded in the ABI PRISM 3130 automated sequencer (Applied Biosystems, USA). The GenBank accession numbers for the complete nucleotide sequence data of the 11 gene segments of strain RVA/Human-wt/BEL/BE5028/2012/G3P[14] are KP258398-KP258408.

### 2.5. Determination of the 5’ and 3’ Ends

The 5’ and 3’ ends were determined to obtain the complete nucleotide sequences by using the single primer amplification method [25]. For this aim the ligation and sequencing procedure was used as described previously [26].

### 2.6. Nucleotide Sequence Analysis

The chromatograms obtained were analysed using Chromas 2.3 (Technelysium, Queensland, Australia), and contigs were prepared by using SeqMan II (DNASTAR, Madison, WI). The sequences were manually corrected and compared with other sequences available in GenBank. The RVA classification tool RotaC 2.0v (rotac.regatools.be) [27], was used to obtain the genotypes of all segments.

### 2.7. Phylogenetic Analysis

For phylogenetic analysis and alignments the Molecular Evolutionary Genetics Analysis (MEGA) 6.0 was used [28]. Identities between the sequences were determined at the nucleotide level (P-distance option). In phylogenetic analyses we opted for maximum likelihood phylogenetic analyses. Bootstrap resampling analysis (500 replicates) was performed to measure the reliability of the tree topologies. For the identification of rearrangements and further comparison between sequences, the bioinformatics tools GeneDoc and JDotter were used [29,30].

## 3. Results

### 3.1. Complete Genome Sequencing of BE5028

The complete nucleotide sequences of the 11 gene segments of the Belgian strain BE5028 were determined by Sanger sequencing. The genotype constellation of BE5028 was compared to RVAs isolated from a rabbit (30–96) and a human (B4106) with a typical lapine genotype constellation, in this manuscript further referred to as lapine and lapine-like RVAs. Comparison was also made with two other G3P[14] RVAs (N5 and RCH272), and K1130027, another RVA strain isolated from a rabbit. It was found that lapine strain BE5028 shared the same constellation with the lapine(-like) strains 30–96 and B4106 (G3-P[14]-I2-R2-C2-M3-A9-N2-T6-E5-H3), whereas it possessed seven differences (VP1, VP2, VP6, NSP2-NSP5) with the Chinese lapine strain N5, five differences (VP3, VP4, VP7, NSP1 and NSP4) with the Dutch G6P[11] strain K1130027, and three differences (VP1, VP2 and NSP4) with the Australian G3P[14] strain RCH272 (Table 1).

**Table 1:** Genotype constellations of completely sequenced lapine(-like) and reference RVA strains. Green, red, and orange indicate Wa-like, DS-1-like, and AU-1-like gene segments, respectively. Violet indicates typical lapine genotypes.

Strain Name	VP7	VP4	VP6	VP1	VP2	VP3	NSP1	NSP2	NSP3	NSP4	NSP5
RVA/Human-tc/USA/Wa/1974/G1P[8]	G1	P[8]	I1	R1	C1	M1	A1	N1	T1	E1	H1
RVA/Human-tc/USA/DS-1/1976/G2P[4]	G2	P[4]	I2	R2	C2	M2	A2	N2	T2	E2	H2
RVA/Human-tc/JPN/AU-1/1982/G3P[9]	G3	P[9]	I3	R3	C3	M3	A3	N3	T3	E3	H3
**RVA/Human-wt/BEL/BE5028/2012/G3P[14]**	**G3**	**P[14]**	**I2**	**R2**	**C2**	**M3**	**A9**	**N2**	**T6**	**E5**	**H3**
RVA/Human-wt/BEL/B4106/2000/G3P[14]	G3	P[14]	I2	R2	C2	M3	A9	N2	T6	E5	H3
RVA/Rabbit-tc/ITA/30-96/1996/G3P[14]	G3	P[14]	I2	R2	C2	M3	A9	N2	T6	E5	H3
RVA/Rabbit-tc/CHN/N5/1992/G3P[14]	G3	P[14]	I17	R3	C3	M3	A9	N1	T1	E3	H2
RVA/Human-wt/AUS/RCH272/2012/G3P[14]	G3	P[14]	I2	R3	C3	M3	A9	N2	T6	E2	H3
RVA/Rabbit-tc/NLD/K1130027/2011/G6P[11]	G6	P[11]	I2	R2	C2	M2	A13	N2	T6	E2	H3

### 3.2. Phylogenetic Analysis

**Figure 1:**
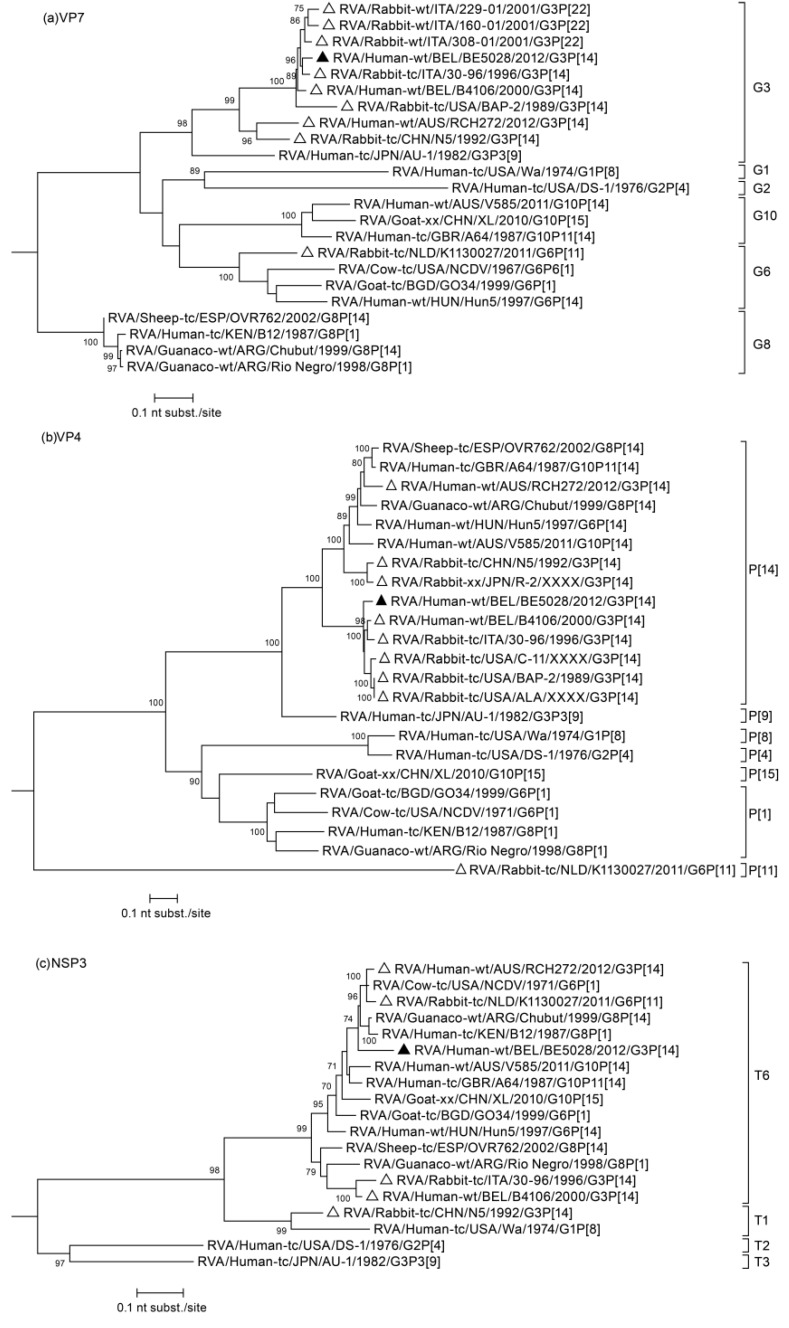

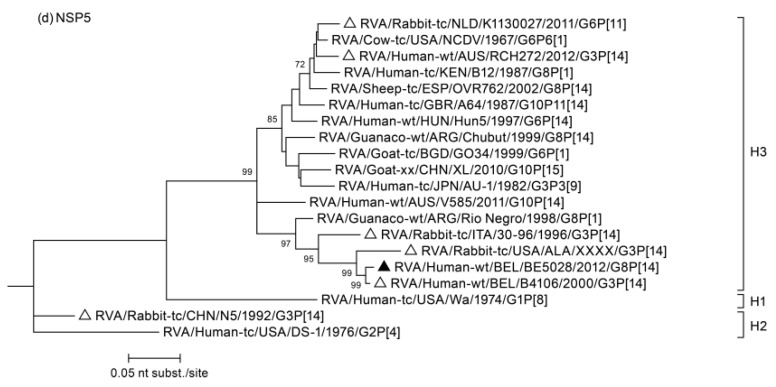
Phylogenetic trees of the BE5028 (**a**) VP7; (**b**) VP4; (**c**) NSP3 and (**d**) NSP5 genes. Bootstrap values (500 replicates) above 70 are shown. Strain BE5028 is indicated by a black triangle, other lapine(-like) RVA strains are indicated with a white triangle.

The sequences of all segments were blasted, closely related sequences were selected and multiple alignments were made for each gene segment together with appropriate reference sequences available in GenBank. The analysis of segment 9 (Figure 1a), which encodes for VP7, showed that BE5028 clustered most closely to lapine strains 30–96 and 308–01, and also to the human lapine-like strain B4106, which was previously demonstrated to have a lapine origin [14]. At the nucleotide level these lapine(-like) strains showed 94.7%–95.9% similarity with BE5028. The phylogenetic analysis of VP4 (shown in Figure 1b) revealed that, as in the phylogenetic tree of VP7, the BE5028 strain clustered closely together with lapine(-like) strains. In particular, it is most closely related with human strain B4106 showing a nucleotide similarity of 94.5%. Interestingly, the NSP3 phylogenetic tree (Figure 1c), showed that the Belgian strain BE5028 shared relatively low genetic relatedness, more specifically 94.0% and 96.4% nucleotide similarity to 30–96 and B4016, the other typical lapine(-like) strains with the T6 genotype. The most closely related strain to BE5028 was the bovine NCDV strain, a tissue culture adapted strain isolated from a cow in the USA in 1971 (92.7% similar at the nucleotide level). The phylogenetic analysis of NSP5 confirmed the H3 genotype as BE5028 was most closely related to the lapine-like B4106 strain, sharing a nucleotide similarity of 98.9% (Figure 1d). For segments VP1, VP2, VP3, VP6, NSP1, NSP2, and NSP4, it was also shown that BE5028 was most closely related to the human lapine-like strain B4106 (Figure S1), with a nucleotide similarity ranging from 96.4% (NSP4) to 99.3% (NSP2).

### 3.3. Duplication in NSP5 Segment

**Figure 2:**
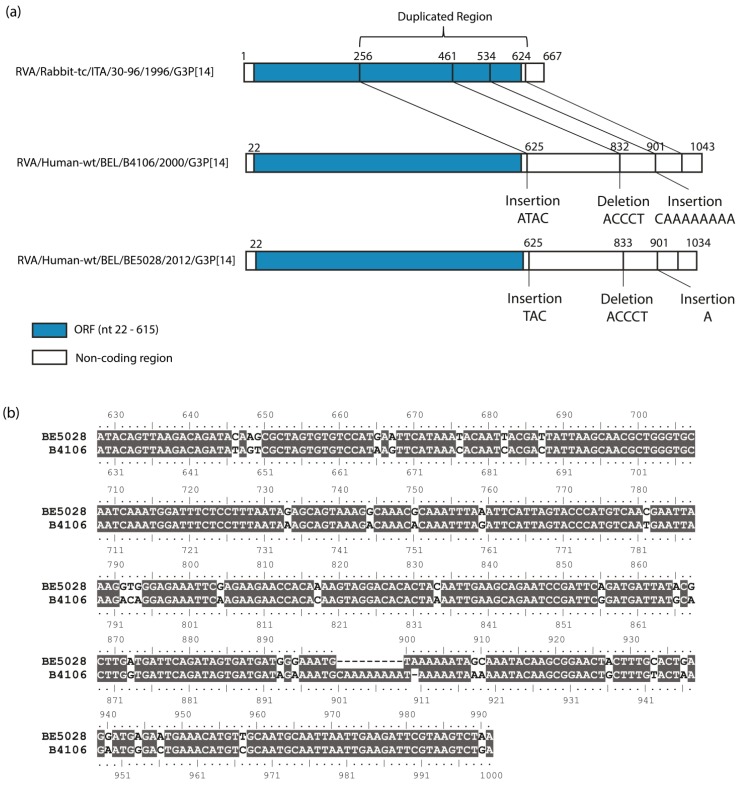
(**a**) A schematic representation of the rearrangements found in the duplicated region of the NSP5 of BE5028, compared to the NSP5 sequence of 30–96 and to the duplicated region of the NSP5 of B4106. The blue region indicates the ORF of NSP5; (**b**) Sequence alignment of the duplicated sequence of BE5028 and B4106 NSP5. Identical residues are shown in grey.

The NSP5 segment of BE5028 was 1034 nt long. The ORF (Open Reading Frame) of NSP5 comprehended 198 amino acids (from nt 22 to nt 615), whereas the ORF of NSP6 included 96 amino acids (from nt 80 to nt 367). Through a dot plot comparison between the lapine rotavirus 30–96 NSP5 segment and the NSP5 gene of the BE5028 strain, a duplication of 368 nucleotides was detected (data not shown), starting after the stop codon of NSP5 and reiterating the NSP5 sequence staring at nucleotide 256. In addition, two small insertions and a deletion were found in the duplicated region. By comparison of both sequences (NSP5 segments of 30–96 and BE5028), it was determined that the first insertion was a triplet (TAC), the deletion involved five nucleotides (ACCCT), while the last insertion was of just one adenine, as shown in Figure 2a. A comparison between the duplicated region of BE5028 and the homologous coding sequence of BE5028, showed a difference of 18.2%. A similar duplication was previously described for the human strain B4106 [14], which was very closely related to the BE5028 strain for most gene segments, except for NSP3. In Figure 2b, a sequence alignment between the duplicated region of BE5028 and B4106 shows the similarity (90.4% at the nucleotide level) between these two Belgian strains, which were isolated 12 years apart in the same Belgian province.

## 4. Discussion

The G3P[14] genotype combination is atypical in humans while it is common in rabbits [13,14,15]. Thus far, G3P[14] RVA strains have only been found twice in humans: strains B4106 and RCH272. The first one caused gastroenteritis in a hospitalized six-year-old child in Belgium in 2000, and was very closely related with a lapine strain (30–96) leading to the hypothesis that strain B4106 was the result of the transmission of a rabbit rotavirus to a human [14]. RCH272 caused gastroenteritis in a 12-year-old child in Australia. Due to the fact that the genome was genetically divergent to previously characterized lapine rotaviruses and the genes were distantly related to human bovine-like and other animal strains, it was hypothesized that RCH272 represents a direct transmission from an unknown host species or was derived by multiple reassortment events in various animal species [24]. BE5028 was found to share the same genotype constellation with both B4106 and 30–96: G3-P[14]-I2-R2-C2-M3-A9-N2-T6-E5-H3, while it showed three genotype differences (VP1, VP2, and NSP4) with RCH272. Phylogenetic analyses of all 11 gene segments showed that BE5028 clustered very closely together with lapine strains and with the lapine-like human B4106 strain, except for NSP3. Although this segment shared the same genotype (T6) with the other lapine and lapine-like strains it was most closely related with an old, previously characterized bovine strain NCDV, suggesting a past reassortment event between bovine and lapine RVA strains. However, there was only 92.7% nucleotide similarity between BE5028 and NCDV, therefore BE5028 could also represent a yet unidentified cluster within the T6 genotype that circulates in the rabbit population. As little is known about Belgian lapine and bovine RVAs, it would be interesting to investigate the rotavirus diversity present in rabbits and cows living in the same geographical area as the children that got infected. As a previous study already showed the possibility for a bovine(-like) RVA strain to infect and circulate in a rabbit population exists [20]. Altogether these findings highlight the need for RVA surveillance in animals as a powerful tool to identify reassortment and interspecies transmission events.

Of particular interest was the detection of a head-to-tail partial duplication of 368 nucleotides in the non-coding region of the NSP5 segment. The pairwise distance between the coding region of BE5028 and the duplicated sequence is 17.9%. This difference is probably due to accumulation of mutations over time in the non-coding region. A highly similar duplication was previously found and described in the lapine-like human strain B4106 [14]. Both BE5028 and B4106 were isolated in Belgium, in the same geographical area (approximately 20 km apart). The coding sequences of BE5028 and B4106 were almost identical (98.9%). Comparison of the duplicated regions of BE5028 and B4106 showed that both duplications start at the same nucleotide (nt 256) and share almost the same length (364 and 372). The duplicated regions of BE5028 and B4106 showed only 35 nucleotide differences, which is little compared to the number of nucleotide differences between BE5028 and 30–96 (65 nucleotide differences) or B4106 and 30–96 (68 nucleotide differences). It can be hypothesized that both duplications originated from the same rearrangement in the same ancestral strain, and over the years underwent different point mutations, insertions and deletions, but kept on circulating in the rabbit population. Rearrangements in NSP5 are more frequent than in other rotavirus genes, although the reason for this is still unknown [2,3,31]. Duplications in the NSP5 segment were also detected previously in rabbits, pigs, and human strains [31,32,33,34,35,36].

Altogether, these findings suggest that the RVA strain BE5028 with a typical lapine genotype constellation was the second lapine-like strain able to cause acute gastroenteritis in a child in Belgium after B4106. The limited number of reports describing interspecies transmission are not restricted to lapine to human transmission. Other examples of studies reporting interspecies transmissions from animal to human include canine, feline, bovine, porcine, and unknown host strains [24,36,37,38,39,40,41]. Interestingly, zoonotic infections often seem to be dead end infections as these rotavirus strains does not seem to be replicated very efficient in a different host [23]. Despite the fact that there is no evidence of human to human transmission of G3P[14] strains, this study indicates that children can develop gastroenteritis after infection with a lapine RVA strain, highlighting the need for continued surveillance of RVAs not only in humans, but also in animals.

## Supplementary Files

Supplementary File 1

Supplementary File 2
