# Supplementary material for: Complete Genome Analysis of a Rabbit Rotavirus Causing Gastroenteritis in a Human Infant
Source: Viruses. 2015 Feb 17;7(2):844–56. doi: 10.3390/v7020844 (PMC4353919; doi:10.3390/v7020844)
Supplement: Supplementary File 1 [file viruses-07-00844-s001.pdf]

Table S1. Primers used for amplification and sequencing of the whole genome of rotavirus strain RVA/Human-wt/BEL/BE5028/2012/G3P[14].

Gene	Primer name	Primer sequence
VP1	LAP_VP1_384R	5'-TGC TGT TGG AAA AAG TTC G -3'
	LAP_VP1_539F	5'-ACT ATG AAG TAA TGA AAG ATA AGC C-3'
	LAP_VP1_2238R	5'-CAC TGT GTG CTT TGT CC -3'
	LAP_VP1_2761R	5'-CAA TTT GAT AGG TTC TGG ATC C -3'
	LAP_VP1_2930F	5'-ACG CTG ATA CGT ATG TCG -3'
VP2	B1711_VP2_2112R	5 -GCA ATT TTA TCT GAG GCA CG -3
	LAP_VP2_137R	5'-TCA GCA ATT TTA ACC TCT TCC-3'
	LAP_VP2_142F	5'-GTG AAA AAA TCG ACA AAA GAG-3'
	LAP_VP2_530F	5'- CAG GTT CTC ACT GAA ATG C-3'
	LAP_VP2_1650R	5'-TCA ACT AGT TGA CCA AGC C -3'
	LAP_VP2_2159R	5'-TCT TTC TAA TTG CAT GTC ACG-3'
	LAP_VP2_2543F	5'-GAC ATC AAA TCT TAC CTT CAC TG-3'
VP3	Au-1_VP3_2348F	5'- TGT TAG TGG AGT TTT AGC GAC -3'
	LAP_VP3_258R	5'-CAA GAG ACT GAA TCT CAA TAG C-3'
	LAP_VP3_599F	5'-GCA CGA CTA TCG AAC AGA G-3'
	LAP_VP3_1971R	5'-TCG ACC TTT TCT ATT GAA TGC-3'
VP4	F05028_VP4_1376F	5'-GCC GGA AGA TTC TCG TTG AT-3'
	D3-16_VP4_165R	5'-ATG ACC CCA ATT GAC TGG-3'
	LAP_VP4_1F	5'-GGC TAT AAA ATG GCT TCT TTG-3'
	LAP_VP4_788F	5'-CCT TGT GGA AAG AAA TGC-3'
	LAP_VP4_1624R	5'-CCA CCG TAG ATT TTA TTC CTG-3'
	LAP_VP4_2068F	5'-ATT CGA AAC TGG AAT CGA TGG-3'
VP6	L338_VP6_1183F	5'-CAG TAG CTT CCA TTA GAA GCA TG-3'
	LAP_VP6_169R	5'-CCA GTT TGG AAC TCA TTT CC-3'
	LAP_VP6_521F	5'-CAT TCA CAC TGA ACA GAT CG-3'
VP7	F05028_VP7_262R	5'-CTG TGT GGA ATT AGC GTA TGC-3'
	F05028_VP7_980F	5'-GCA CCA CAA ACT GAA AGG ATG-3'
NSP1	E403_NSP1_20F	5'-TCT TGT GTT AGC CAT GGC G-3'
	F05028_NSP1_174R	5'-AGT TGA CAG CAT TCG ACA CAC-3'
	LAP_NSP1_1370F	5'-AAT CAA TGG TGC AAC GTG-3'
	LAP_NSP1_1497R	5'-CAG AAT CTG AGA TCA GAA GC-3'
NSP2	260-97_NSP2_459F	5'-ATT GAG ACA ACT GCC ACT GC-3'
	LAP_NSP2_910F	5'-ACT GTT CCA AAA GAT GAA GC-3'
	MG6_NSP2_155R	5'-TGT CCA CTT TTG CTG TCA AC-3'
NSP3	B383_NSP3_486R	5'-ACC TCA ACT TCA CCA CGT TC-3'
	F05028_NSP3_1494F	5'-CCA TCT TCA CAT GAC CCT CT-3'
NSP4	LAP_NSP4_357R	5'-TTT CTC TGA CTA CTC TGT CC-3'
	LAP_NSP4_559F	5'-CAT CAT TGT GAG AGG TTG G-3'
NSP5	06-030_NSP5_511F	5'-GAT GAT TCA GAT AGT GAT GAT GG-3'
	LAP_NSP5_314R	5'-CGT GAT TGT GTT GAT GAA TCC-3'
