# Supplementary material for: Complete Genome Analysis of a Rabbit Rotavirus Causing Gastroenteritis in a Human Infant
Source: Viruses. 2015 Feb 17;7(2):844–56. doi: 10.3390/v7020844 (PMC4353919; doi:10.3390/v7020844)
Supplement: Supplementary File 2 [file viruses-07-00844-s002.pdf]

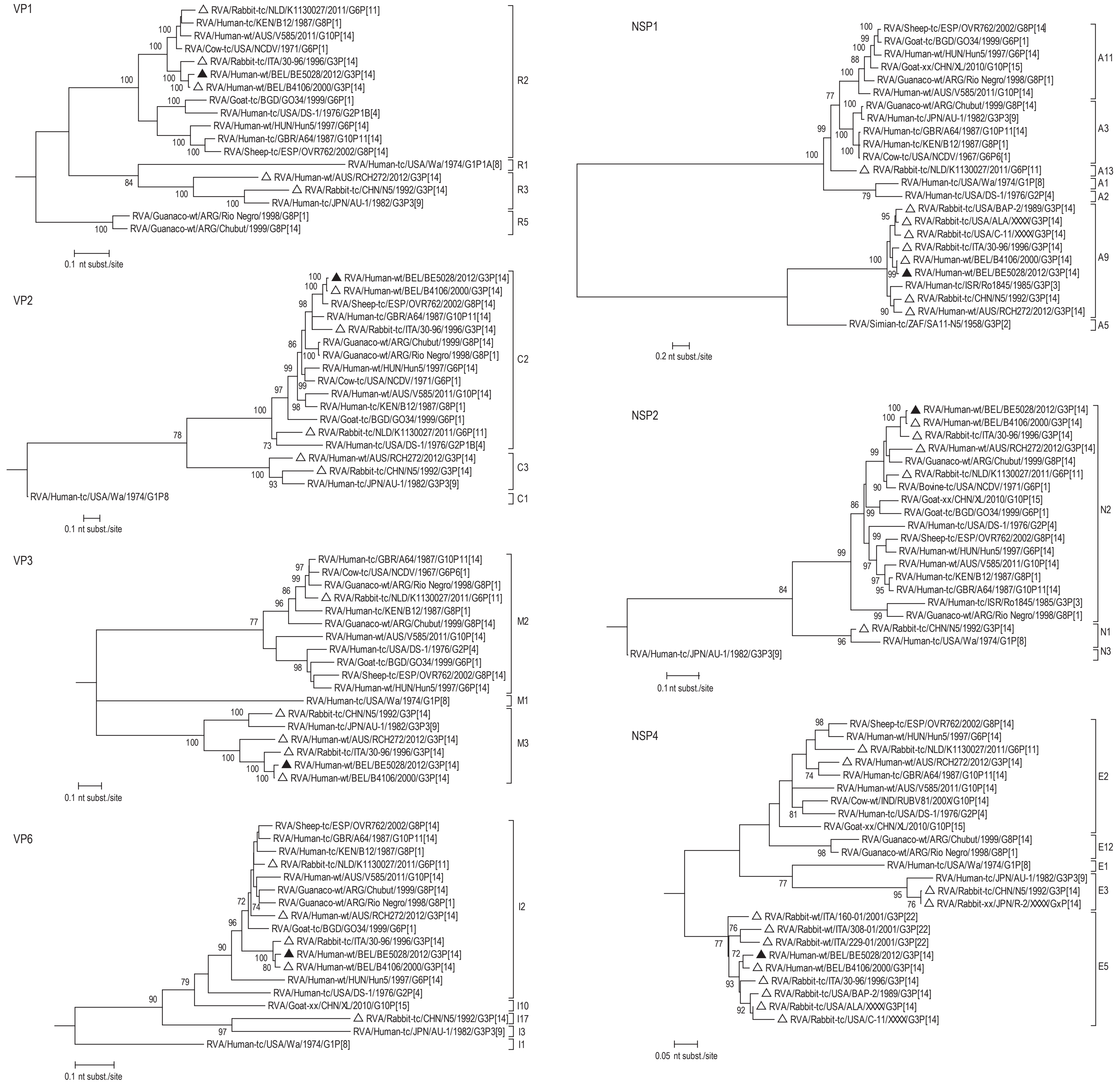

Figure S1. Phylogenetic trees of the BE5028 VP1, VP2, VP3, VP6, NSP1, NSP2 and NSP4 genes. Bootstrap values (500 replicates) above 70 are shown. Strain BE5028 is indicated by a black triangle, other lapine(-like) RVA strains are indicated with a white triangle.
